# Going viral: a review of replication-selective oncolytic adenoviruses

**DOI:** 10.18632/oncotarget.5116

**Published:** 2015-08-07

**Authors:** Christopher Larson, Bryan Oronsky, Jan Scicinski, Gary R. Fanger, Meaghan Stirn, Arnold Oronsky, Tony R. Reid

**Affiliations:** ^1^ University of California at San Diego (UCSD), Moores Cancer Center, La Jolla, CA, USA; ^2^ EpicentRx, Inc., Mountain View, CA, USA; ^3^ InterWest Partners, Menlo Park, CA, USA

**Keywords:** oncology, immunotherapy, oncolytic virus, adenovirus

## Abstract

Oncolytic viruses have had a tumultuous course, from the initial anecdotal reports of patients having antineoplastic effects after natural viral infections a century ago to the development of current cutting-edge therapies in clinical trials. Adenoviruses have long been the workhorse of virotherapy, and we review both the scientific and the not-so-scientific forces that have shaped the development of these therapeutics from wild-type viral pathogens, turning an old foe into a new friend. After a brief review of the mechanics of viral replication and how it has been modified to engineer tumor selectivity, we give particular attention to ONYX-015, the forerunner of virotherapy with extensive clinical testing that pioneered the field. The findings from those as well as other oncolytic trials have shaped how we now view these viruses, which our immune system has evolved to vigorously attack, as promising immunotherapy agents.

## INTRODUCTION

Multiple forces aside from fundamental science can influence the development of any therapeutic, and oncolytic viruses are no exception. Before delving into the scientific aspects, we review the cultural evolution of virotherapy and the enthusiasm and skepticism that accompanied its development, regardless of how well founded those outlooks may have been.

Early virus discoveries date back to 1892 when Dimitrii Ivanovsky observed that the agent of tobacco mosaic disease passed through porcelain filters that retained bacteria leading to the term “filterable agent”, which was first used assuming that these were small bacteria. The name virus arose from the Latin word meaning slimy liquid or poison and was originally used to describe any infectious agent, including the agent of tobacco mosaic disease, tobacco mosaic virus. In 1898 Marcus Beijerinck concluded that the pathogen must be a distinctive agent, and in the same year Friedrich Loeffler and Paul Frosch (former students of Robert Koch), found that the causative agent of foot-and-mouth disease was filterable (the first animal virus) and in 1901 Walter Reed identified the Yellow fever virus as the first human virus. From these humble and inauspicious beginnings, the field of virotherapy arose.

Through the 1950's-1970's, years before the advent of the World Wide Web, one clinical concept that quite literally “went viral” was the use of pathogenic viruses to treat, and possibly cure, cancer. It is more than a little ironic that viruses, which, at times, pose a significant health threat, have been and continue to be seen in this hopeful—and righteous—light. The foundation for this anthropomorphized “good virus” belief was laid as far back as 1904 when Dr. George Dock, a University of Michigan Hematology Professor, described a 42-year-old woman with acute leukemia who experienced a temporary remission after a presumed infection with influenza in 1896 [1] though at the time the relationship between the influenza virus and the infectious syndrome it caused was not established. Similarly, in 1912 a cervical cancer patient bitten by a dog developed extensive tumor necrosis following administration of a live attenuated rabies virus for post-exposure prophylaxis [2]. In addition, spontaneous clinical remissions have been observed in Hodgkin lymphoma [3] and Burkitt's lymphoma [4] after natural infections with measles virus.

Many more of these types of anecdotal observations were reported where spontaneous remissions and regressions of tumors occurred in the setting of naturally acquired viral infections [5], possibly due to stimulation of an anti-tumor immune response rather than direct oncolysis [6]. They prompted hundreds of clinical trials initiated between 1950-1970 to investigate the treatment of cancer by administration of different viruses that included hepatitis, West Nile, yellow fever, dengue fever, Uganda and adenoviruses, as enthusiasm for their potential to cure the disease reached a fever pitch [5]. They all used wild-type viruses because these trials predated the availability of modern molecular biology techniques to modify their genomes.

Since these trials were not performed according to current clinical standards, interpreting the data is difficult. Moreover, to say that stringent quality control and testing were lacking in these trials is a gross understatement: in some cases patients were inoculated with viruses from previous patients [7, 8], or the same patients received multiple injections of different viruses (e.g., West Nile, Newcastle, vaccinia). The consequences from this haphazard and uncoordinated administration in terms of safety were predictably disastrous [9], with a high risk of encephalitis from West Nile, Uganda, dengue, and yellow fever.

On the other hand, adenovirus (or adenoidal-pharyngeal-conjunctival virus as it was formerly known) rose to prominence in cervical cancer clinical trials with a demonstration of safety with mild flu-like symptoms being the main adverse event [10], and apparent efficacy in the form marked tumor necrosis or liquefaction of tumors. However, the enthusiasm for oncolytic adenoviruses was as short-lived [11, 12] as antitumor responses lasted only months and did not translate into an overall survival benefit [13] even when there was extensive necrosis within the treated tumors [14], unlike the promising new chemotherapy agents introduced in the 1970s. Consequently, interest waned.

The 1970s were a heady time in oncology: Nixon's presidentially promoted War on Cancer [15] in 1971 injected fresh urgency and momentum into the discovery of clinically active molecules, resulting in an ever-expanding therapeutic arsenal of new combination chemotherapy regimens at the bedside which promised to revolutionize the treatment of the disease. In the optimism for combination chemotherapies that characterized the zeitgeist, these hard-to-control viruses with a poor track record were ‘mothballed’ and forgotten, leaving the field to drift into semi-obsolescence. From foe to friend and back to foe again, viruses were an unlikely “*deus ex machina”*, having suddenly appeared on the scene as the potential answer to cancer and then, just as suddenly, faded away again.

The latency period lasted until the mid-1980s when, thanks to a revolution in molecular biology, virotherapy made a comeback with the development of adeno and retroviral vectors for transgene delivery in different indications including cancer [16]. However, the scientific consensus soon oscillated from excessively optimistic to excessively pessimistic after gene transfer, by and large, was not effectively demonstrated. In addition, a lack of understanding of how gene transfer vectors interacted with the host coupled with the lack of suitable animal models to fully understand the biology further dampened enthusiasm [17].

In the early 2000s the field suffered yet another setback of sorts when the worldwide rights to dl1520 (ONYX-015), a genetically engineered adenovirus with multiple completed Phase I and Phase II trials, were sold by Onyx to the Chinese company, Shanghai Sunway Biotech, who halted development of the virus midway during Phase III. This led to the mistaken impression that ONYX-015 was an ineffective therapeutic strategy [18]; in fact, the virus likely would have met its primary overall survival (OS) endpoint if the Phase III had been allowed to continue. Indeed, the Chinese State Food and Drug Administration approved H101 (Oncorine), an oncolytic adenovirus similar to ONYX-015, for use in combination with chemotherapy for the treatment of late-stage refractory nasopharyngeal cancer in November, 2005 [19]. The corporate backstory [20] was that in 1995 Warner Lambert entered into a partnership with Onyx to develop the virus. However, in 2000, through the twists and turns of corporate strategy, Warner Lambert merged with Pfizer who backed out of the Onyx deal—a 40 million dollar commitment for completion of Phase III trials—on the basis of their skepticism about the Phase I and II clinical trial data and lack of enthusiasm for virotherapy in general. With the split from Warner Lambert/Pfizer and the loss of the 40 million dollars, Onyx decided to divest itself of the virus and cash out, focusing instead on its other partnered asset: the tyrosine kinase inhibitor sorafenib (Nexavar®) co-funded by Bayer and subsequently approved for hepatocellular carcinoma (HCC) in 2005.

As it turned out, this fateful decision to sell ONYX-015, which may have made financial sense at the time, stigmatized virotherapy as a whole [21], despite the seeming validation from the 2005 regulatory approval of H101 (Oncorine), a closely related adenovirus developed by Shanghai Sunway Biotech, for the treatment of head and neck cancers in China. It was hard to shake the perception of failure, whatever the actual facts, and virotherapy was largely dismissed, overlooked or ignored [22] until very recently when interest rebounded with the advent of highly effective immunotherapeutic agents. The potential for synergistic and rational combinations of checkpoint inhibitors, radiotherapy and adoptive cell therapy may finally mark a decisive turning point in the renewed interest of oncolytic viruses, due to the real potentiation of the cellular immune response, discussed below. Moreover, to enhance efficacy, the oncolytic viruses may be “armed” with additional therapeutic genes which can drive a systemic antitumor immune response. In the meantime, multiple viruses including herpesvirus, reovirus, polio virus, rhabdovirus, parvovirus and vaccinia have quietly entered clinical trials [23, 24].

The ideal oncolytic virus (OV) is easily produced, easily manipulated, selectively lytic to cancer cells, systemically deliverable and, above all, safely administerable with low intrinsic pathogenicity—criteria, which, by and large, are met by adenoviridae. By contrast, vaccinia and HSV [25] with large dsDNA genomes that encode hundreds of proteins, require multiple deletions for safety and specificity while retroviruses pose the theoretical risk of insertional mutagenesis i.e. insertion of viral genomes into the host genome.

This article reviews the biology of oncolytic adenoviruses, summarizes the data from clinical trials and finally explores future directions in combination with immunotherapy. Non-adenoviral oncolytics have also undergone extensive clinical development and entered late-phase clinical trials, most notably herpesvirus (talimogene laherparepvec, previously called oncovex) [26–31], vaccinia (pexa-vec, previously JX-594), [32–36] and reovirus (reolysin), [37–46] while others are emerging as potential therapeutics. Our focus on adenoviruses in this review is in no way meant to detract from their major contributions to the field of virotherapy.

## BIOLOGY OF HUMAN ADENOVIRUS

Adenovirus biology, which serves as the basis for the rational design of conditionally replicative adenoviruses (Ads), is briefly reviewed here. In humans, more than 50 different serotypes of adenovirus have been discovered [47]. On the basis of sequence homology and their ability to agglutinate red blood cells [48], these serotypes are divided into six species or subgroups (labeled A-F), most of which are responsible for benign respiratory tract and gastrointestinal infections [49]. The two most commonly described and developed for oncolytic therapy, 2 and 5 (group C), are non-oncogenic [50] since the viruses replicate episomally (i.e., extrachromosomally) without host genome insertion [51].

**Figure 1:**
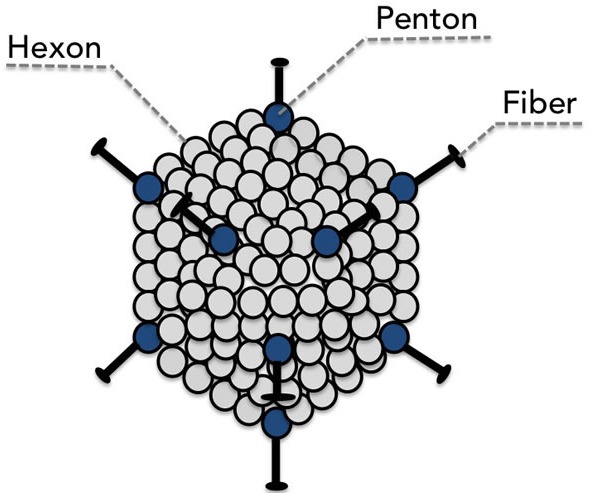
The major capsid proteins fiber, penton, and hexon are the principle mediators of binding to the host cell

Adenoviridae are a family of icosahedral, non-enveloped (meaning that they possess a protein capsid instead of a lipid membrane) viruses with an approximately 30-40 kb linear double-stranded DNA genome [51]. The capsid proteins, in particular the hexon, penton base, and fiber, (see Figure 1) are principally responsible for host-receptor binding primarily through the coxsackie and adenovirus receptor (CAR) and virus internalization [52]. These capsid proteins disassemble inside the cell, resulting in the subsequent nuclear import of the viral genome [53] for commencement of viral transcription.

The adenovirus replicative cycle is divided into two phases: early (E1-E4) and late (L1-L5) (See Table 1). The first expressed viral transcription unit is E1A, which contains four highly conserved regions (CR1-CR4) [54]. The two major E1A mRNA gene products, 12S and 13S, have a variety of functions that include promotion of epigenetic alterations through their interaction with chromatin remodeling protein p400 and the histone acetyltransferases (HATs) p300/CBP [55] and dissociation of retinoblastoma (Rb) from its complex with E2F, which in turn activates E2F-mediated transcription of the other adenovirus early transcription units (E1B, E2, E3 and E4) involved in DNA synthesis and S-phase induction [56].

**Table 1:** Functions of Adenovirus Genes

E = Early (before replication, <8 hours) L = Late (after replication, >12 hours)	
Gene	Function
E1A	Modifies the function of key host and viral regulatory proteins such as retinoblastoma (Rb) and the chromatin remodeling protein p400
E2	Encodes the proteins for viral DNA replication
E3	Modulates host defense mechanisms
E4, E1B	Progression to late phase
L1-L5	Capsid proteins

**Figure 2:**
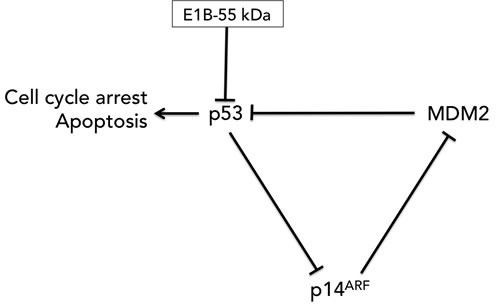
The p53-MDM2-p14^ARF^ feedback loop MDM2 negatively regulates p53 by promoting p53 degradation. p14^ARF^ (p19^ARF^ in the mouse) binds to and sequesters MDM2, thereby preventing MDM2 from targeting p53 for degradation while p53 negatively regulates p14^ARF^ expression. E1B-55kDa binds to and degrades p53. The removal of this gene in the ONYX-015 virus did not necessarily relieve p53 inhibition due to the presence of the other two components of the feedback loop in the tumor: MDM2 and p14^ARF^.

E2F, the host cell transcription factor that activates the transcription of the adenovirus E2 gene, induces p53-dependent apoptosis [57]. In order to allow E2F to transcribe these viral genes while avoiding its induction of p53-dependent apoptosis, adenoviruses produce a 55K E1b protein which binds to p53 [58] and exports it to the cytoplasm for degradation, thereby keeping the host cell alive long enough for productive infection [59]. The ONYX-015 virus, which will be discussed in more detail later in this review, is E1B-55k-deleted [60]. In theory, as McCormick originally postulated, that would make it a p53-selective cancer therapy. However, McCormick [61] and others [62]later reported that ONYX-015 [60] replicates efficiently in p53^+^ tumor cells [63], possibly due to the tumors inhibiting p53 activity through other mechanisms such as overexpression of the endogenous p53 inhibitor, Mdm2, or the loss of p14^ARF^, which downregulates Mdm2 (Figure 2).

E1B is transcribed shortly after E1A and encodes the 55K protein, mentioned above, that inhibits the function of p53 as well as a 19K protein, a homolog of cellular Bcl-2, which prevents bax- and bak-mediated mitochondrial apoptosis. The E2 region encodes three proteins required for replication of the viral genome: DNA polymerase, preterminal protein, and the 72-kDa single-stranded DNA-binding protein [64].

E3, dispensable for adenoviral infection *in vitro*, encodes proteins that function to subvert the immune response. These proteins include a 19-kDa glycoprotein (gp19) which downregulates class I major histocompatibility complex-mediated antigen presentation to cytotoxic T-lymphocytes, thereby preventing T cell recognition of infected cells [65]. Three other proteins 10.4K, 14.5K, and 14.7K [66], protect cells from the lytic effects of tumor necrosis factor-α (TNF-α). Because E3 is nonessential, it is often deleted to allow for insertion of foreign DNA (approximately 3.3 kb).

E4 produces transcripts for six proteins named after their open reading frames: ORF1, ORF2, ORF3, ORF4, ORF6 and ORF6/7. These viral proteins are involved in DNA replication, transcription, apoptosis, host cell protein shutoff and regulation of cell cycle signaling [67]. ORF6 [68] cooperates with E1b-55K, discussed earlier, both to inhibit p53 and to induce the preferential transport of viral late mRNAs from the nucleus to the cytoplasm [69].

Late after infection, the major late promoter (MLP) initiates transcription from the so-called major late transcription unit [70]. Its transcripts, subdivided into five late regions (L1-L5), mostly encode the structural proteins needed for progeny virus, e.g. penton base (L2), hexon (L3) and fiber (L5) [71].

## CONDITIONALLY REPLICATIVE ADENOVIRUSES (CRADS)

From an understanding of adenoviral biology, two broad types of replication-competent oncolytic adenoviruses or conditionally replicative Ad (CRAds) were developed to restrict the cytocidal effect to tumors. The first type of CRAd involved modification of a viral gene sequence important for efficient viral replication in normal cells but dispensable or expendable in tumor cells [72]. In the second type of CRAd, tissue/tumor specific promoters were inserted to restrict viral replication to deregulated tumors with the ability to activate these promoters; in normal tissues where the promoter is inactive no viral transcription results, so infection is a dead end. Examples include the human telomerase reverse transcriptase (hTERT)-regulated adenovirus [73], active in multiple tumor types, and prostate specific antigen (PSA)-regulated adenovirus CG7870 developed for the treatment of prostate carcinoma [74]. Those approaches both act after the step of viral entry into the cell: the virus is not modified to selectively enter cancerous cells rather than normal cells, but is modified so it cannot carry out a productive infection to release progeny except in cancerous cells. Modifications to the fiber protein leading to selective entry into cancer cells have also been described [75] and have made it into clinical trials [76], but are generally used in combination with one of the previously mentioned types of modification to confer additional specificity.

As the first engineered adenovirus to enter clinical trials, ONYX-015 is the standard bearer for the whole CRAd field and has been closely followed by other E1b-55k deleted viruses with similar efficacy and toxicity profiles. This class of viruses was designed around deletion of the 800 bp E1b-55k gene, and this was the sole modification in ONYX-015 that did not carry any therapeutic transgenes. In theory, lacking a functional E1B-55 kDa for p53 degradation, the adenovirus should have only replicated in cells with wild-type p53 but not in cells where p53 was mutated or defective. In reality, ONYX-015 replication was p53-independent, likely due to the complexity of the p53-MDM2-p14ARF feedback loop (see Figure 2).

In fact, as O'Shea *et al* have demonstrated, the mechanism of tumor selectivity was related to the presence of the protein Y-Box Binding Factor 1 (YB-1) [77], variably expressed in cancer cells but not found in normal tissue. According to O'Shea, the multifunctional E1B-55 kDa protein mediates export of late adenoviral mRNA in normal cells as well as p53 inhibition [77]. YB1 substitutes for the mRNA export function of E1B-55K only in tumors (although not all tumors are YB1^+^), leading to cancer cell-restricted ONYX-015 replication. In retrospect, this proposed mechanism of action helps to explain the ONYX-015 clinical trial data and suggests future strategies to optimize and improve therapeutic outcomes.

## RESULTS FROM CLINICAL TRIALS WITH ONYX-015 (DL1520)

The advent of viral therapy brought concerns about its safety when retroviral integration causing activation of the proto-oncogene LMO2 led to leukemia in patients treated for SCID [78] and after the death of a patient with ornithine transcarbamylase deficiency treated with adenoviral gene therapy [79, 80]. Such dramatic adverse events have not been observed in cancer patients treated with adenoviruses, and lack of chromosomal integration (and more importantly killing of infected cells) mitigates the risks that integration entails. In Phase I and II trials, ONYX-015 was safe and well tolerated; even at the highest dose of 3 × 10^11^ plaque forming units (PFU) no dose-limiting toxicities were reached [81, 82] by intravenous, intratumoral or hepatic artery administration. The most common reported adverse effect was short-lived flu-like symptoms [83].

The evaluation of efficacy was more complex. RECIST response rates as a single agent were disappointingly low in its trials that predated the development of immune related response criteria for pseudoprogressive changes. Pseudoprogression, i.e. therapy-mediated tumor swelling, which can transiently occur before a tumor later regresses, has been associated and recognized with the checkpoint inhibitors ipilimumab and nivolumab [84], whole brain radiotherapy (WBRT) [85], imatinib in GIST [86] and the Phase II pan-epigenetic inhibitor, RRx-001 [87] where the kinetic responses to these agents are typically slower than with standard chemotherapy.

**Figure 3:**
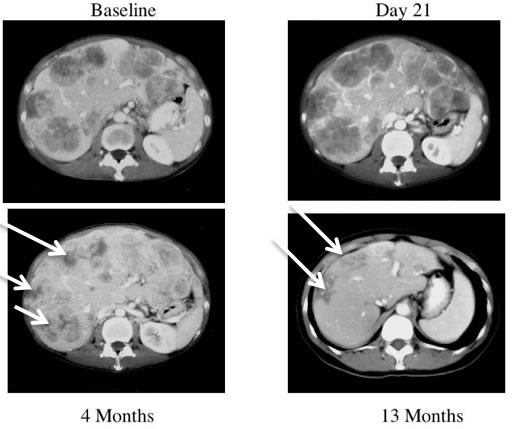
CT scans of a patient receiving ONYX-015 This patient received treatment with ONYX-015 on days 1 and 8 and CT scans are shown from baseline, Day 21, 4 months and 13 months. The baseline CT scan of this patient demonstrates extensive disease in all lobes of the liver. The CT on day 21 demonstrates therapy-related enlargement of the tumor masses so-called pseudoprogression. The patient continued with monthly treatment with ONYX-015 in combination with 5-FU/leucovorin and improvement of the tumor masses (white arrows) was observed at 4 months. The masses (white arrows) have largely resolved by 13 months [88].

Similarly, Reid *et al* [88] reported a pattern of acute tumor enlargement followed by regression of tumor size after intra-hepatic injection of ONYX-015 in combination with 5-FU/leucovorin for hepatic colorectal metastases in 11 of 24 patients (46%), suggestive of an ipilimumab-like pseudoprogression, and predictive for improved survival (Figure 3) A phase II trial of talimogene laherparepvec (then called Oncovex), an attenuated herpesvirus that expresses GM-CSF, showed delayed responses occurring up to ten months after starting treatment and often preceded by apparent tumor progression [29]. These trials suggest that pseudoprogression may be a common theme in oncolytic virotherapy [89].

While ONYX-015 was a pioneering agent, there is room for potential improvement through further viral genetic modification: E1b-55k is a multifunctional protein with pleiotropic properties including mRNA transport and its deletion renders the virus YB-1-dependent, which affects replication efficiency and lytic activity since not all tumors express YB-1. However, with an intact E1b-55k gene, tumors remain fully permissive and susceptible to Ad infection. For example, adenovirus 5 mutants dl922-947 and D24, which retain E1b-55k and carry a mutation in the conserved region 2 (CR-2) of the E1A gene that abolishes binding of E1A to Rb protein and prevents release of the E2F transcription factor, have demonstrated significantly greater potency compared to ONYX-015 both *in vitro* and *in vivo* [90].

ONYX-015 has cancer-selective replication due to the E1B-55k deletion but is otherwise unmodified, and in particular it did not carry a therapeutic “cargo” transgene. Other adenoviruses with various cargoes have been tested in the clinic with varying degrees of success. But when interpreting responses to virally delivered cytokines, one must keep in mind that the cargo gene is not the only immunologic factor in play. The viral packaging system itself will elicit an innate host response even without viral replication or even cellular expression of foreign proteins [91, 92], and replicating viruses would stimulate the same innate and adaptive immune response as a natural viral infection.

While systemically administered interleukin-2 can induce durable disease control in about 10% of treated patients, a replication-defective adenovirus delivering the interleukin-2 gene induced responses in some tumors that were directly injected with the virus but not in distant metastases [93]. In the replication-defective adenovirus TNFerade, TNF alpha was driven by a radiation inducible promoter to achieve high local TNF levels without detectable levels in the blood and the associated toxicities of systemic TNF [94]. Early phase trials of TNFerade gave encouraging results with melanoma [95], but a phase III trial recently found no survival benefit in patients with pancreatic cancer [96] and it was not established whether the radiation inducible promoter in that non-replicating (and therefore not strictly oncolytic) virus actually led to significant TNF expression in patients. CD154 has shown promise - in a phase I/IIa trial of patients with urothelial carcinoma of the bladder, intravesicular adenovirus carrying CD154 led to absence of tumor on cystectomy in 3 out of 5 patients with high risk cancer with plans for cystectomy, and to tumor shrinkage in 1 out of 3 patients who had stage Ta tumors [97]. A replication-defective adenovirus delivering a modified form of CD154 with increased membrane stability was injected into lymph nodes of patients with CLL, and in a dose-escalation study it reduced lymphocytosis, lymphadenopathy, or splenomegaly in most patients [98].

GM-CSF was identified as promising for cancer immunotherapy in preclinical work and has emerged as the leading cytokine in the virotherapy field. The most notable preclinical study of GM-CSF was an *in-vivo* screen by Dranoff and colleagues: tumor cell lines carrying various immunostimulatory agents were used to immunize mice, and vaccination with the GM-CSF expressing cells provided the greatest protection against later engraftment when mice were challenged with the parental tumor cell lines [99]. Cell-mediated anti-tumor responses were seen in humans when a replication competent tumor-selective adenovirus was armed with GM-CSF and injected intratumorally to patients with various metastatic cancers, and generated MHC I dependent T-cell activity against the tumor-associated antigen survivin while inducing clinical responses (tumor regression or stabilization) in 7 of 15 patients [100]. ONCOS-102 (Ad5/3-D24-GMCSF) with triple orphan status for mesothelioma, soft tissue sarcoma and ovarian cancer in the U.S. and Europe showed evidence of immune priming on biopsies in Phase I clinical trials [101] (discussed below). ONCOS-102 is similar to Ad5 D24 but with the following modifications: 1) The Ad 5 fiber knob domain has been replaced with an Ad 3 fiber knob that binds to cells via a Coxsackie Adenovirus Receptor (CAR)-independent pathway, which is often downregulated in advanced tumors [89] and 2) It is armed with an immunostimulatory granulocyte-macrophage colony stimulating factor (GMCSF) transgene in the E3 region.

## IMMUNOGENIC EFFECTS OF VIROTHERAPY AND POTENTIAL FOR COMBINATION WITH IMMUNOTHERAPY

One of the criticisms of OVs has been that their efficacy is limited to directly treated tumors as intravenously administered viruses fail to reach distant metastatic disease due to off-target tissue trapping (e.g. in liver or spleen) and they are rapidly cleared [102] by neutralizing antibodies. Furthermore, the immune response is rapidly mobilized to clear the viral infection, which on the one hand is beneficial because it limits systemic spread and prevents the potential for widespread toxicity, but on the other is detrimental due to the inhibition of therapeutic efficacy. Strategies to suppress the immune response and rapid viral clearance include PEGylation [103], cell or nanoparticle carriers [104], modification of the tumor vasculature [105] and/or transient immunosuppression [106].

Fortunately, evidence indicates that oncolytic virotherapy, like the systemic or abscopal effects of radiotherapy [107], is not restricted to direct tumor cytolysis and apoptosis but also primes an immune response against distant lesions due to cell death, production of cytokines, and release of tumor antigens. One editorial about oncolytic viruses posits the question, which it doesn't actually go on to answer, about whether OVs are really “direct tumor killers or simply immune adjuvants” [108]. Indeed, T cell and dendritic cell (DC) activation and stimulation of innate and adaptive antitumor immunity have been reported with adenovirus [109] and other oncolytic therapies [27, 36].

For this reason, oncolytic adenoviral therapy is particularly suitable for combination with other immunostimulatory/immunomodulatory treatments including cytokines, Treg-depleting chemotherapies like cyclophosphamide, DC-based vaccines like sipuleucel-T (Provenge^®^) and, of course, the checkpoint inhibitors such as cytotoxic T-lymphocyte antigen 4 (CTLA-4) and programmed death 1 receptor (PD-1), which “release the brakes” on the adaptive antitumor immune response [110]. This was demonstrated in one small trial where metronomic cyclophosphamide, which selectively depletes Tregs, augmented the activity of an oncolytic adenovirus carrying GM-CSF [111], and trials combining talimogene laherparepvec with checkpoint inhibitors are planned.

Furthermore, insertion of immunostimulatory transgenes (e.g. ligand traps, cytokines, costimulatory molecules etc.) from oncolytic viruses is also likely to potentiate antitumor activity, and incorporation of GM-CSF into viruses is now commonplace.

## FUTURE DIRECTIONS

According to an ancient Arab proverb, “The enemy of my enemy is my friend.” It is by this logic that viruses, a threat to human welfare and existence since time immemorial, have been conscripted to fight the War on Cancer, due to their inherent tumor-tropic and anticancer properties recognized since the turn of the 20^th^ century. Through the seemingly impossible magic of genetic engineering, a plethora of viruses have been reengineered into weapons of (tumor) mass destruction. Viruses are particularly inimical to cancer cells because the immunosuppression that shields tumors from the innate and adaptive immune system also increases their susceptibility to pathogenic attack: like “no-go” neighborhoods with high levels of crime that are largely off-limits to the police, the immune-protected enclave of the tumor allows viral replication to proceed largely unchecked. Of the variety of infectious viral species developed as virotherapy agents, adenoviruses have emerged as one of the most promising because they are intrinsically oncolytic for tumor cells while minimally toxic to normal non-transformed cells.

**Figure 4:**
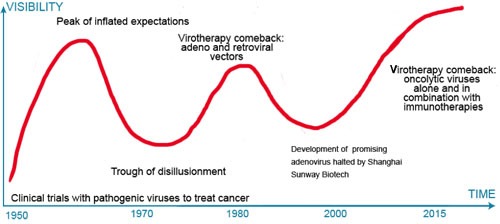
Times are *trough* all over The expectation (hype)-disappointment cycle in virotherapy. Throughout its history, oncolytic virotherapy (OV) has been characterized by over-enthusiasm followed by pessimism and apathy when expectations were not met. With the immunotherapy revolution interest in OV has rebounded.

Despite the potential of oncolytic adenoviruses, especially in combination with checkpoint inhibitors or other immunotherapy, we should learn from the boom-bust cycles of hype and disappointment in the decades of the 1950's, 1960's and 1980's and carefully resist the temptation to characterize these OVs as “magic bullets” since no one strategy by itself is—or will likely ever be— a panacea—a single “one shot” solution to the riddle, wrapped in a mystery, inside an enigma that is cancer. Unrealistic, unattainable expectations will inevitably lead to disillusionment and another bursting of the bubble for the virotherapy field (see Figure 4), which can, in turn, undermine its potential and lead to a withdrawal of interest or support.

Although oncolytic adenoviruses have meaningful single agent antitumor activity, complementary mechanisms of action and nonoverlapping toxicity profiles make them a more ideal radiation, chemotherapy, kinase inhibitor, and/or immunotherapy partner than a stand-alone treatment. For example, the combination of metronomic cyclophosphamide and the oncolytic adenovirus, ONCOS-102, in a Phase I solid tumor trial resulted in a significant reduction of T_regs_ in the presence of a Th1-like response—T_reg_ infiltration not only correlates with poor prognosis [112] but also compromises the efficacy of immunotherapies. Moreover, because OVs stimulate an anti-tumor immune response, combination with checkpoint inhibitors will undoubtedly improve therapeutic efficacy. Several research groups including the Reid lab are evaluating the combination of OVs with traditional chemotherapy agents, multikinase inhibitors and CTLA-4 and PD1 inhibitors.

Autophagy-inducing agents like temozolomide have also been reported to potentiate the activity of oncolytic viruses [113]. In addition to temozolomide, another intriguing potential combination is with the experimental pan-epigenetic inhibitor, radiosensitizer [114] and autophagy inducer, RRx-001, which has demonstrated promising activity without systemic toxicity in Phase I and Phase II metastatic colorectal cancer and brain metastasis (+ whole brain radiation) studies [115].

## CONCLUSION

Despite the plethora of therapeutic combinations with actual or theoretical benefit, there is room to improve the single agent activity of adenoviruses. Tumors overexpress growth factors, cytokines, transcription factors, stress signals, and oncogenic stimuli, which may supply deleted viral mutants with missing functions. The implication of this tumor ‘helper function’ is that point deletions of a few base pairs, rather than whole genes, may be all that are required to impair replication in normal tissue, while permitting near-wild-type levels of replication and expression in cancer cells. Viruses pack tremendous complexity into a small genome, often with overlapping reading frames and proteins carrying out multiple functions as exemplified by E1B-19k mediating both p53 neutralization and RNA export. Therefore, with regard to oncolytic adenoviral engineering, less is more. While many oncolytic viruses carry GM-CSF as a therapeutic gene, other approaches using different genes are certainly possible and it is tempting to speculate about how they could be strategically used to fight cancer. If a checkpoint inhibitor given systemically proves to be synergistic with an oncolytic virus, then why not make the virus itself deliver a checkpoint inhibitor to be produced within the tumor? The tumor microenvironment is characterized by aberrant expression of cytokines promoting tumor progression like TGF-β [116], and viruses may be a tool particularly well suited to manipulate the tumor microenvironment itself. They offer the possibility to produce therapeutic agents directly within treated tumors, turning the problem of delivering agents to a dense tumor with high interstitial pressure completely around and leading to greater intratumoral activity and fewer systemic side effects.

Possibilities for the future are brighter than ever: wild-type adenoviruses, a common cause of respiratory, diarrheal and conjunctival disease, may infect about a billion people worldwide every year [117], but perhaps one day not too far in the future genetically engineered adenoviruses will treat billions more as a therapy not only for cancer but also potentially many other diseases.

## Abbreviations

APC: adenoidal-pharyngeal-conjunctival virus
CAR: coxsackie and adenovirus receptor
CR-2: conserved region 2
CRAds: conditionally replicative Ad
CTLA-4: cytotoxic T-lymphocyte antigen 4
DC: dendritic cell
GMCSF: granulocyte-macrophage colony stimulating factor
HATs: histone acetyltransferases
hTERT: human telomerase reverse transcriptase
MLP: major late promoter
MLTU: major late transcription unit
OS: overall survival
OTC: ornithine transcarbamylase
OV: Oncolytic virus
PD-1: programmed death 1 receptor
PFU: plaque forming units
PSA: prostate specific antigen
Rb: retinoblastoma
TNF-α: tumor necrosis factor-α
WBRT: whole brain radiotherapy
YB-1: Y-Box Binding Factor 1

## References

[1] Dock G (1904). The influence of complicating diseases upon leukaemia. Am J Med Sci.

[2] Pack G (1950). Note of the experimental use of rabies vaccine for melanomatosis. Arch Dermatol Syphilol.

[3] Taqi AM, Abdurrahman MB, Yakubu AM, Fleming AF (1981). Regression of Hodgkin's disease after measles. Lancet.

[4] Bluming AZ, Ziegler JL (1971). Regression of Burkitt's lymphoma in association with measles infection. Lancet.

[5] Kelly E, Russell SJ (2007). History of Oncolytic Viruses: Genesis to Genetic Engineering. Mol Ther.

[6] Ring CJ (2002). Cytolytic viruses as potential anti-cancer agents. J Gen Virol.

[7] Newman W, Southam CM (1954). Virus treatment in advanced cancer; a pathological study of fifty-seven cases. Cancer.

[8] Southam CM, Moore AE (1954). Induced virus infections in man by the Egypt isolates of West Nile virus. Am J Trop Med Hyg.

[9] Singh PK, Doley J, Kumar GR, Sahoo AP, Tiwari AK (2012). Oncolytic viruses & their specific targeting to tumour cells. Indian J Med Res.

[10] Huebner RJ, Bell JA, Rowe WP, Ward TG, Suskind RG, Hartley JW, Paffenbarger RS (1955). Studies of adenoidal-pharyngeal-conjunctival vaccines in volunteers. J Am Med Assoc.

[11] Zielinski T, Jordan E (1969). [Remote results of clinical observation of the oncolytic action of adenoviruses on cervix cancer]. Nowotwory.

[12] Rowe WP, Huebner RJ, Gilmore LK, Parrott RH, Ward TG (1953). Isolation of a cytopathogenic agent from human adenoids undergoing spontaneous degeneration in tissue culture. Proc Soc Exp Biol Med.

[13] Georgiades J, Zielinski T, Cicholska A, Jordan E (1959). Research on the oncolytic effect of APC viruses in cancer of the cervix uteri; preliminary report. Biul Inst Med Morsk Gdansk.

[14] Huebner RJ, Rowe WP, Schatten WE, Smith RR, Thomas LB (1956). Studies on the use of viruses in the treatment of carcinoma of the cervix. Cancer.

[15] Oronsky B, Carter CA, Mackie V, Scicinski J, Oronsky A, Oronsky N, Caroen S, Parker C, Lybeck M, Reid T (2014). The war on cancer: a military perspective. Front Oncol.

[16] Hemminki A, Alvarez RD (2002). Adenoviruses in oncology: a viable option?. BioDrugs.

[17] Orkin SH, Motulsky AG, NIH (1995). Report and recommendations of the panel to assess the NIH investment in research on gene therapy.

[18] Jia H, Kling J (2006). China offers alternative gateway for experimental drugs. Nat Biotechnol.

[19] Liu TC, Kirn D (2008). Gene therapy progress and prospects cancer: oncolytic viruses. Gene Ther.

[20] McCormick F (2007). Success and failure on the ras pathway. Cancer Biol Ther.

[21] Garber K (2006). China approves world's first oncolytic virus therapy for cancer treatment. J Natl Cancer Inst.

[22] Theodore F, Theodore F (2012). Methods in Enzymology. Methods in Enzymology.

[23] Friedman GK, Cassady KA, Beierle EA, Markert JM, Gillespie GY (2012). Targeting pediatric cancer stem cells with oncolytic virotherapy. Pediatr Res.

[24] Wollmann G, Ozduman K, van den Pol AN (2012). Oncolytic virus therapy for glioblastoma multiforme: concepts and candidates. Cancer J.

[25] Van Etten JL, Lane LC, Dunigan DD (2010). DNA viruses: the really big ones (giruses). Annu Rev Microbiol.

[26] Liu BL, Robinson M, Han ZQ, Branston RH, English C, Reay P, McGrath Y, Thomas SK, Thornton M, Bullock P, Love CA, Coffin RS (2003). ICP34. 5 deleted herpes simplex virus with enhanced oncolytic, immune stimulating, and anti-tumour properties. Gene Ther.

[27] Kaufman HL, Kim DW, DeRaffele G, Mitcham J, Coffin RS, Kim-Schulze S (2010). Local and distant immunity induced by intralesional vaccination with an oncolytic herpes virus encoding GM-CSF in patients with stage IIIc and IV melanoma. Ann Surg Oncol.

[28] Hu JC, Coffin RS, Davis CJ, Graham NJ, Groves N, Guest PJ, Harrington KJ, James ND, Love CA, McNeish I, Medley LC, Michael A, Nutting CM, Pandha HS, Shorrock CA, Simpson J (2006). A phase I study of OncoVEXGM-CSF, a second-generation oncolytic herpes simplex virus expressing granulocyte macrophage colony-stimulating factor. Clin Cancer Res.

[29] Senzer NN, Kaufman HL, Amatruda T, Nemunaitis M, Reid T, Daniels G, Gonzalez R, Glaspy J, Whitman E, Harrington K, Goldsweig H, Marshall T, Love C, Coffin R, Nemunaitis JJ (2009). Phase II clinical trial of a granulocyte-macrophage colony-stimulating factor-encoding, second-generation oncolytic herpesvirus in patients with unresectable metastatic melanoma. J Clin Oncol.

[30] Harrington KJ, Hingorani M, Tanay MA, Hickey J, Bhide SA, Clarke PM, Renouf LC, Thway K, Sibtain A, McNeish IA, Newbold KL, Goldsweig H, Coffin R, Nutting CM (2010). Phase I/II study of oncolytic HSV GM-CSF in combination with radiotherapy and cisplatin in untreated stage III/IV squamous cell cancer of the head and neck. Clin Cancer Res.

[31] Andtbacka RH, Kaufman HL, Collichio F, Amatruda T, Senzer N, Chesney J, Delman KA, Spitler LE, Puzanov I, Agarwala SS, Milhem M, Cranmer L, Curti B, Lewis K, Ross M, Guthrie T (2015). Talimogene Laherparepvec Improves Durable Response Rate in Patients With Advanced Melanoma. J Clin Oncol.

[32] Mastrangelo MJ, Maguire HC, Eisenlohr LC, Laughlin CE, Monken CE, McCue PA, Kovatich AJ, Lattime EC (1999). Intratumoral recombinant GM-CSF-encoding virus as gene therapy in patients with cutaneous melanoma. Cancer Gene Ther.

[33] Hwang TH, Moon A, Burke J, Ribas A, Stephenson J, Breitbach CJ, Daneshmand M, De Silva N, Parato K, Diallo JS, Lee YS, Liu TC, Bell JC, Kirn DH (2011). A mechanistic proof-of-concept clinical trial with JX-594, a targeted multi-mechanistic oncolytic poxvirus, in patients with metastatic melanoma. Mol Ther.

[34] Park BH, Hwang T, Liu TC, Sze DY, Kim JS, Kwon HC, Oh SY, Han SY, Yoon JH, Hong SH, Moon A, Speth K, Park C, Ahn YJ, Daneshmand M, Rhee BG (2008). Use of a targeted oncolytic poxvirus, JX-594, in patients with refractory primary or metastatic liver cancer: a phase I trial. Lancet Oncol.

[35] Breitbach CJ, Burke J, Jonker D, Stephenson J, Haas AR, Chow LQ, Nieva J, Hwang TH, Moon A, Patt R, Pelusio A, Le Boeuf F, Burns J, Evgin L, De Silva N, Cvancic S (2011). Intravenous delivery of a multi-mechanistic cancer-targeted oncolytic poxvirus in humans. Nature.

[36] Heo J, Reid T, Ruo L, Breitbach CJ, Rose S, Bloomston M, Cho M, Lim HY, Chung HC, Kim CW, Burke J, Lencioni R, Hickman T, Moon A, Lee YS, Kim MK (2013). Randomized dose-finding clinical trial of oncolytic immunotherapeutic vaccinia JX-594 in liver cancer. Nat Med.

[37] Strong JE, Coffey MC, Tang D, Sabinin P, Lee PW (1998). The molecular basis of viral oncolysis: usurpation of the Ras signaling pathway by reovirus. EMBO J.

[38] Kelly KR, Espitia CM, Mahalingam D, Oyajobi BO, Coffey M, Giles FJ, Carew JS, Nawrocki ST (2012). Reovirus therapy stimulates endoplasmic reticular stress, NOXA induction, and augments bortezomib-mediated apoptosis in multiple myeloma. Oncogene.

[39] Vidal L, Pandha HS, Yap TA, White CL, Twigger K, Vile RG, Melcher A, Coffey M, Harrington KJ, DeBono JS (2008). A phase I study of intravenous oncolytic reovirus type 3 Dearing in patients with advanced cancer. Clin Cancer Res.

[40] Gollamudi R, Ghalib MH, Desai KK, Chaudhary I, Wong B, Einstein M, Coffey M, Gill GM, Mettinger K, Mariadason JM, Mani S, Goel S (2010). Intravenous administration of Reolysin, a live replication competent RNA virus is safe in patients with advanced solid tumors. Invest New Drugs.

[41] Galanis E, Markovic SN, Suman VJ, Nuovo GJ, Vile RG, Kottke TJ, Nevala WK, Thompson MA, Lewis JE, Rumilla KM, Roulstone V, Harrington K, Linette GP, Maples WJ, Coffey M, Zwiebel J (2012). Phase II trial of intravenous administration of Reolysin((R)) (Reovirus Serotype-3-dearing Strain) in patients with metastatic melanoma. Mol Ther.

[42] Morris DG, Feng X, DiFrancesco LM, Fonseca K, Forsyth PA, Paterson AH, Coffey MC, Thompson B (2013). REO-001: A phase I trial of percutaneous intralesional administration of reovirus type 3 dearing (Reolysin(R)) in patients with advanced solid tumors. Invest New Drugs.

[43] Forsyth P, Roldan G, George D, Wallace C, Palmer CA, Morris D, Cairncross G, Matthews MV, Markert J, Gillespie Y, Coffey M, Thompson B, Hamilton M (2008). A phase I trial of intratumoral administration of reovirus in patients with histologically confirmed recurrent malignant gliomas. Mol Ther.

[44] Lolkema MP, Arkenau HT, Harrington K, Roxburgh P, Morrison R, Roulstone V, Twigger K, Coffey M, Mettinger K, Gill G, Evans TR, de Bono JS (2011). A phase I study of the combination of intravenous reovirus type 3 Dearing and gemcitabine in patients with advanced cancer. Clin Cancer Res.

[45] Comins C, Spicer J, Protheroe A, Roulstone V, Twigger K, White CM, Vile R, Melcher A, Coffey MC, Mettinger KL, Nuovo G, Cohn DE, Phelps M, Harrington KJ, Pandha HS (2010). REO-10: a phase I study of intravenous reovirus and docetaxel in patients with advanced cancer. Clin Cancer Res.

[46] Karapanagiotou EM, Roulstone V, Twigger K, Ball M, Tanay M, Nutting C, Newbold K, Gore ME, Larkin J, Syrigos KN, Coffey M, Thompson B, Mettinger K, Vile RG, Pandha HS, Hall GD (2012). Phase I/II trial of carboplatin and paclitaxel chemotherapy in combination with intravenous oncolytic reovirus in patients with advanced malignancies. Clin Cancer Res.

[47] Benko M, Harrach B, Russell W, Van Regenmortel M, Fauquet C, Bishop D (2000). Virus Taxonomy; Seventh Report of the International Committee on Taxonomy of Viruses. Virus Taxonomy; Seventh Report of the International Committee on Taxonomy of Viruses.

[48] Shenk T, Fields B, Knipe D, Howley P (1996). Adenoviridae: the viruses and their replication. Virology.

[49] Purkayastha A, Ditty SE, Su J, McGraw J, Hadfield TL, Tibbetts C, Seto D (2005). Genomic and bioinformatics analysis of HAdV-4, a human adenovirus causing acute respiratory disease: implications for gene therapy and vaccine vector development. J Virol.

[50] Cook JL, Lewis AM (1984). Differential NK cell and macrophage killing of hamster cells infected with nononcogenic or oncogenic adenovirus. Science.

[51] Williams PD, Ranjzad P, Kakar SJ, Kingston PA (2010). Development of viral vectors for use in cardiovascular gene therapy. Viruses.

[52] Schoggins JW, Falck-Pedersen E (2006). Fiber and penton base capsid modifications yield diminished adenovirus type 5 transduction and proinflammatory gene expression with retention of antigen-specific humoral immunity. J Virol.

[53] Trotman LC, Mosberger N, Fornerod M, Stidwill RP, Greber UF (2001). Import of adenovirus DNA involves the nuclear pore complex receptor CAN/Nup214 and histone H1. Nat Cell Biol.

[54] Chakraborty AA, Tansey WP (2009). Adenoviral E1A function through Myc. Cancer Res.

[55] Frisch SM, Mymryk JS (2002). Adenovirus-5 E1A: paradox and paradigm. Nat Rev Mol Cell Biol.

[56] Nemajerova A, Talos F, Moll UM, Petrenko O (2008). Rb function is required for E1A-induced S-phase checkpoint activation. Cell Death Differ.

[57] Dynlacht BD (2005). E2F and p53 make a nice couple: converging pathways in apoptosis. Cell Death Differ.

[58] Berk AJ (2005). Recent lessons in gene expression, cell cycle control, and cell biology from adenovirus. Oncogene.

[59] Rao L, Debbas M, Sabbatini P, Hockenbery D, Korsmeyer S, White E (1992). The adenovirus E1A proteins induce apoptosis, which is inhibited by the E1B 19-kDa and Bcl-2 proteins. Proc Natl Acad Sci U S A.

[60] Harada JN, Berk AJ (1999). p53-Independent and -dependent requirements for E1B-55K in adenovirus type 5 replication. J Virol.

[61] McCormick F (2000). ONYX-015 selectivity and the p14ARF pathway. Oncogene.

[62] Goodrum FD, Ornelles DA (1998). p53 status does not determine outcome of E1B 55-kilodalton mutant adenovirus lytic infection. J Virol.

[63] Au T, Thorne S, Korn WM, Sze D, Kirn D, Reid TR (2007). Minimal hepatic toxicity of Onyx-015: spatial restriction of coxsackie-adenoviral receptor in normal liver. Cancer Gene Ther.

[64] Nagata K, Guggenheimer RA, Hurwitz J (1983). Specific binding of a cellular DNA replication protein to the origin of replication of adenovirus DNA. Proc Natl Acad Sci U S A.

[65] Burgert HG, Kvist S (1987). The E3/19K protein of adenovirus type 2 binds to the domains of histocompatibility antigens required for CTL recognition. EMBO J.

[66] Deryckere F, Burgert HG (1996). Tumor necrosis factor alpha induces the adenovirus early 3 promoter by activation of NF-kappaB. J Biol Chem.

[67] Halbert DN, Cutt JR, Shenk T (1985). Adenovirus early region 4 encodes functions required for efficient DNA replication, late gene expression, and host cell shutoff. J Virol.

[68] Harada JN, Shevchenko A, Shevchenko A, Pallas DC, Berk AJ (2002). Analysis of the adenovirus E1B-55K-anchored proteome reveals its link to ubiquitination machinery. J Virol.

[69] Gonzalez RA, Flint SJ (2002). Effects of mutations in the adenoviral E1B 55-kilodalton protein coding sequence on viral late mRNA metabolism. J Virol.

[70] Farley DC, Brown JL, Leppard KN (2004). Activation of the early-late switch in adenovirus type 5 major late transcription unit expression by L4 gene products. J Virol.

[71] Kochanek S, Schwab M (2009). Adenovirus. Encyclopedia of Cancer.

[72] Abou El Hassan MA, van der Meulen-Muileman I, Abbas S, Kruyt FA (2004). Conditionally replicating adenoviruses kill tumor cells via a basic apoptotic machinery-independent mechanism that resembles necrosis-like programmed cell death. J Virol.

[73] Kanaya T, Kyo S, Hamada K, Takakura M, Kitagawa Y, Harada H, Inoue M (2000). Adenoviral expression of p53 represses telomerase activity through down-regulation of human telomerase reverse transcriptase transcription. Clin Cancer Res.

[74] Small EJ, Carducci MA, Burke JM, Rodriguez R, Fong L, van Ummersen L, Yu DC, Aimi J, Ando D, Working P, Kirn D, Wilding G (2006). A phase I trial of intravenous CG7870, a replication-selective, prostate-specific antigen-targeted oncolytic adenovirus, for the treatment of hormone-refractory, metastatic prostate cancer. Mol Ther.

[75] Coughlan L, Vallath S, Gros A, Gimenez-Alejandre M, Van Rooijen N, Thomas GJ, Baker AH, Cascallo M, Alemany R, Hart IR (2012). Combined fiber modifications both to target alpha(v)beta(6) and detarget the coxsackievirus-adenovirus receptor improve virus toxicity profiles *in vivo* but fail to improve antitumoral efficacy relative to adenovirus serotype 5. Hum Gene Ther.

[76] Kim KH, Dmitriev IP, Saddekni S, Kashentseva EA, Harris RD, Aurigemma R, Bae S, Singh KP, Siegal GP, Curiel DT, Alvarez RD (2013). A phase I clinical trial of Ad5/3-Delta24, a novel serotype-chimeric, infectivity-enhanced, conditionally-replicative adenovirus (CRAd), in patients with recurrent ovarian cancer. Gynecol Oncol.

[77] O'Shea CC, Johnson L, Bagus B, Choi S, Nicholas C, Shen A, Boyle L, Pandey K, Soria C, Kunich J, Shen Y, Habets G, Ginzinger D, McCormick F (2004). Late viral RNA export, rather than p53 inactivation, determines ONYX-015 tumor selectivity. Cancer Cell.

[78] Hacein-Bey-Abina S, Von Kalle C, Schmidt M, McCormack MP, Wulffraat N, Leboulch P, Lim A, Osborne CS, Pawliuk R, Morillon E, Sorensen R, Forster A, Fraser P, Cohen JI, de Saint Basile G, Alexander I (2003). LMO2-associated clonal T cell proliferation in two patients after gene therapy for SCID-X1. Science.

[79] Raper SE, Chirmule N, Lee FS, Wivel NA, Bagg A, Gao GP, Wilson JM, Batshaw ML (2003). Fatal systemic inflammatory response syndrome in a ornithine transcarbamylase deficient patient following adenoviral gene transfer. Mol Genet Metab.

[80] Savulescu J (2001). Harm, ethics committees and the gene therapy death. J Med Ethics.

[81] Reid T, Warren R, Kirn D (2002). Intravascular adenoviral agents in cancer patients: lessons from clinical trials. Cancer Gene Ther.

[82] Ganly I, Kirn D, Eckhardt G, Rodriguez GI, Soutar DS, Otto R, Robertson AG, Park O, Gulley ML, Heise C, Von Hoff DD, Kaye SB (2000). A phase I study of Onyx-015, an E1B attenuated adenovirus, administered intratumorally to patients with recurrent head and neck cancer. Clin Cancer Res.

[83] Griesenbach U (2007). Progress and Prospects: Gene Therapy Clinical Trials (Part 2). Gene Ther.

[84] Wolchok JD, Hoos A, O'Day S, Weber JS, Hamid O, Lebbe C, Maio M, Binder M, Bohnsack O, Nichol G, Humphrey R, Hodi FS (2009). Guidelines for the evaluation of immune therapy activity in solid tumors: immune-related response criteria. Clin Cancer Res.

[85] Parvez K, Parvez A, Zadeh G (2014). The diagnosis and treatment of pseudoprogression, radiation necrosis and brain tumor recurrence. Int J Mol Sci.

[86] Werewka-Maczuga A, Osinski T, Chrzan R, Buczek M, Urbanik A (2011). Characteristics of computed tomography imaging of gastrointestinal stromal tumor (GIST) and related diagnostic problems. Pol J Radiol.

[87] Reid T, Infante J, Burris H, Scribner C, Knox S, Oronsky B, Stephens J, Scicinski J (2013). Activity observed in a phase I dose escalation trial of the hypoxia-activated, NO prodrug, RRx-001. J Clin Oncol.

[88] Reid TR, Freeman S, Post L, McCormick F, Sze DY (2005). Effects of Onyx-015 among metastatic colorectal cancer patients that have failed prior treatment with 5-FU/leucovorin. Cancer Gene Ther.

[89] Hemminki A (2014). Oncolytic immunotherapy: where are we clinically?. Scientifica (Cairo).

[90] Heise C, Hermiston T, Johnson L, Brooks G, Sampson-Johannes A, Williams A, Hawkins L, Kirn D (2000). An adenovirus E1A mutant that demonstrates potent and selective systemic anti-tumoral efficacy. Nat Med.

[91] Shayakhmetov DM, Di Paolo NC, Mossman KL (2010). Recognition of virus infection and innate host responses to viral gene therapy vectors. Mol Ther.

[92] Smith JS, Xu Z, Tian J, Palmer DJ, Ng P, Byrnes AP (2011). The role of endosomal escape and mitogen-activated protein kinases in adenoviral activation of the innate immune response. PLoS One.

[93] Dummer R, Rochlitz C, Velu T, Acres B, Limacher JM, Bleuzen P, Lacoste G, Slos P, Romero P, Urosevic M (2008). Intralesional adenovirus-mediated interleukin-2 gene transfer for advanced solid cancers and melanoma. Mol Ther.

[94] Rasmussen H, Rasmussen C, Lempicki M, Durham R, Brough D, King CR, Weichselbaum R (2002). TNFerade Biologic: preclinical toxicology of a novel adenovector with a radiation-inducible promoter, carrying the human tumor necrosis factor alpha gene. Cancer Gene Ther.

[95] MacGill RS, Davis TA, Macko J, Mauceri HJ, Weichselbaum RR, King CR (2007). Local gene delivery of tumor necrosis factor alpha can impact primary tumor growth and metastases through a host-mediated response. Clin Exp Metastasis.

[96] Herman JM, Wild AT, Wang H, Tran PT, Chang KJ, Taylor GE, Donehower RC, Pawlik TM, Ziegler MA, Cai H, Savage DT, Canto MI, Klapman J, Reid T, Shah RJ, Hoffe SE (2013). Randomized phase III multi-institutional study of TNFerade biologic with fluorouracil and radiotherapy for locally advanced pancreatic cancer: final results. J Clin Oncol.

[97] Malmstrom PU, Loskog AS, Lindqvist CA, Mangsbo SM, Fransson M, Wanders A, Gardmark T, Totterman TH (2010). AdCD40L immunogene therapy for bladder carcinoma—the first phase I/IIa trial. Clin Cancer Res.

[98] Castro JE, Melo-Cardenas J, Urquiza M, Barajas-Gamboa JS, Pakbaz RS, Kipps TJ (2012). Gene immunotherapy of chronic lymphocytic leukemia: a phase I study of intranodally injected adenovirus expressing a chimeric CD154 molecule. Cancer Res.

[99] Dranoff G, Jaffee E, Lazenby A, Golumbek P, Levitsky H, Brose K, Jackson V, Hamada H, Pardoll D, Mulligan RC (1993). Vaccination with irradiated tumor cells engineered to secrete murine granulocyte-macrophage colony-stimulating factor stimulates potent, specific, and long-lasting anti-tumor immunity. Proc Natl Acad Sci U S A.

[100] Cerullo V, Pesonen S, Diaconu I, Escutenaire S, Arstila PT, Ugolini M, Nokisalmi P, Raki M, Laasonen L, Sarkioja M, Rajecki M, Kangasniemi L, Guse K, Helminen A, Ahtiainen L, Ristimaki A (2010). Oncolytic adenovirus coding for granulocyte macrophage colony-stimulating factor induces antitumoral immunity in cancer patients. Cancer Res.

[101] Majumder M, Kumar A, Heckman C, Kankainen M, Pesonen S, Jäger E, Karbach J, Joensuu T, Kairemo K, Partanen K, Alanko T, Hemminki A, Backman C, Dienel K, von Euler M, Hakonen T (2014). Gene expression analysis of tumors demonstrates an induction of Th1 type immune response following intratumoral administration of ONCOS-102 in refractory solid tumor patients. Journal for Immunotherapy of Cancer.

[102] Ferguson MS, Lemoine NR, Wang Y (2012). Systemic delivery of oncolytic viruses: hopes and hurdles. Adv Virol.

[103] O'Riordan CR, Lachapelle A, Delgado C, Parkes V, Wadsworth SC, Smith AE, Francis GE (1999). PEGylation of adenovirus with retention of infectivity and protection from neutralizing antibody *in vitro* and *in vivo*. Hum Gene Ther.

[104] Bolhassani A, Javanzad S, Saleh T, Hashemi M, Aghasadeghi MR, Sadat SM (2014). Polymeric nanoparticles: potent vectors for vaccine delivery targeting cancer and infectious diseases. Hum Vaccin Immunother.

[105] Wojton J, Kaur B (2010). Impact of tumor microenvironment on oncolytic viral therapy. Cytokine Growth Factor Rev.

[106] Dhar D, Toth K, Wold WS (2014). Cycles of transient high-dose cyclophosphamide administration and intratumoral oncolytic adenovirus vector injection for long-term tumor suppression in Syrian hamsters. Cancer Gene Ther.

[107] Siva S, MacManus MP, Martin RF, Martin OA (2015). Abscopal effects of radiation therapy: a clinical review for the radiobiologist. Cancer Lett.

[108] Bell J (2014). Oncolytic viruses: immune or cytolytic therapy?. Mol Ther.

[109] Koski A, Kangasniemi L, Escutenaire S, Pesonen S, Cerullo V, Diaconu I, Nokisalmi P, Raki M, Rajecki M, Guse K, Ranki T, Oksanen M, Holm SL, Haavisto E, Karioja-Kallio A, Laasonen L (2010). Treatment of cancer patients with a serotype 5/3 chimeric oncolytic adenovirus expressing GMCSF. Mol Ther.

[110] Errington F, Steele L, Prestwich R, Harrington KJ, Pandha HS, Vidal L, de Bono J, Selby P, Coffey M, Vile R, Melcher A (2008). Reovirus activates human dendritic cells to promote innate antitumor immunity. J Immunol.

[111] Cerullo V, Diaconu I, Kangasniemi L, Rajecki M, Escutenaire S, Koski A, Romano V, Rouvinen N, Tuuminen T, Laasonen L, Partanen K, Kauppinen S, Joensuu T, Oksanen M, Holm SL, Haavisto E (2011). Immunological effects of low-dose cyclophosphamide in cancer patients treated with oncolytic adenovirus. Mol Ther.

[112] Curiel TJ, Coukos G, Zou L, Alvarez X, Cheng P, Mottram P, Evdemon-Hogan M, Conejo-Garcia JR, Zhang L, Burow M, Zhu Y, Wei S, Kryczek I, Daniel B, Gordon A, Myers L (2004). Specific recruitment of regulatory T cells in ovarian carcinoma fosters immune privilege and predicts reduced survival. Nat Med.

[113] Liikanen I, Ahtiainen L, Hirvinen ML, Bramante S, Cerullo V, Nokisalmi P, Hemminki O, Diaconu I, Pesonen S, Koski A, Kangasniemi L, Pesonen SK, Oksanen M, Laasonen L, Partanen K, Joensuu T (2013). Oncolytic adenovirus with temozolomide induces autophagy and antitumor immune responses in cancer patients. Mol Ther.

[114] Ning S, Bednarski M, Oronsky B, Scicinski J, Saul G, Knox SJ (2012). Dinitroazetidines are a novel class of anticancer agents and hypoxia-activated radiation sensitizers developed from highly energetic materials. Cancer Res.

[115] Reid T, Dad S, Korn R, Oronsky B, Knox S, Scicinski J (2014). Two Case Reports of Resensitization to Previous Chemotherapy with the Novel Hypoxia-Activated Hypomethylating Anticancer Agent RRx-001 in Metastatic Colorectal Cancer Patients. Case Rep Oncol.

[116] Derynck R, Goeddel DV, Ullrich A, Gutterman JU, Williams RD, Bringman TS, Berger WH (1987). Synthesis of messenger RNAs for transforming growth factors alpha and beta and the epidermal growth factor receptor by human tumors. Cancer Res.

[117] Smith JG, Wiethoff CM, Stewart PL, Nemerow GR (2010). Adenovirus. Curr Top Microbiol Immunol.

